# Diversity and composition of root-associated fungal communities in critically small population of *Cypripedium subtropicum*

**DOI:** 10.3389/fpls.2026.1783975

**Published:** 2026-06-03

**Authors:** Mei Hua, Hong Jiang, Ruiling Yuan, Jijun Kong

**Affiliations:** Key Laboratory for Conservation of Rare, Endangered & Endemic Forest Plants, State Forestry Administration/Yunnan Key Laboratory of Biodiversity of Gaoligong Mountain, Yunnan Academy of Forestry and Grassland, Kunming, China

**Keywords:** critically small population wild plants, *Cypripedium subtropicum*, diversity, endangered species conservation, fungal community, orchids

## Abstract

*Cypripedium subtropicum* is a critically small population wild orchid. Understanding the composition and diversity of its root- associated fungal communities is essential for its conservation and artificial propagation. Illumina Novaseq high- throughput sequencing was used to analyze the diversity and composition of fungal communities in the root-endosphere and rhizosphere soil of *C. subtropicum* collected from different regions, habitats, and population types in Yunnan Province. The number of amplicon sequence variants (ASVs) differed by only 0.33% between root-endosphere and rhizosphere soil samples. A total of 34,564 ASVs were detected in root-associated samples from Wenshan Prefecture (Maguan, Malipo, Xichou), accounting for 87.76% of the total ASVs across all samples. Fungal richness of root-associated samples in *C. subtropicum* was highest in Wenshan, followed by Baoshan, Nujiang, and Kunming. Three fungal ASVs were shared between root-endosphere and rhizosphere soil across 120 samples from 12 habitats. Fungal community composition and diversity differed significantly between root-endosphere and rhizosphere soil samples. Samples from Wenshan showed the highest species richness, evenness, and diversity, whereas those from Kunming exhibited the lowest. In Malipo County, fungal abundance and diversity did not differ significantly between wild and introduced populations, but both were significantly higher than in seed-propagated populations in Kunming. Notable regional variations in dominant fungi were observed: Mortierella dominated Wenshan samples, *Archaeorhizomyces* dominated Nujiang samples, *Ilyonectria* dominated Baoshan samples, and *Fusarium* dominated Kunming cultivated samples. NMDS analysis showed Wenshan samples aggregated closely, while other regions’ samples were dispersed. Core fungal species including *Mortierella* sp., *Fusarium solani*, and *Sebacina sp*., which were distributed across all regions. This study reveals clear regional and habitat- driven patterns in root- associated fungal communities of *C. subtropicum*. The findings provide a reliable scientific basis for the conservation and artificial propagation of this endangered orchid.

## 1 Introduction

*Cypripedium subtropicum* is a perennial herb of the family Orchidaceae, genus *Cypripedium*, and taxonomically belongs to the section *Subtropicum* within the subfamily Cypripedioideae. This species was first formally described by Chen and Lang based on specimens collected from Motuo, Xizang, and is characterized by terminal multiflowered racemes, long-stalked staminodes, and seeds with hair-like exotesta (Chen and Lang, 1986). As the only *Cypripedium* species native to subtropical forests in Asia, *C. subtropicum* exhibits highly unique ecological traits: it is evergreen, with plants reaching up to 1.5 m in height; it possesses rhizomes and fleshy roots; its stems are erect and covered with short pubescence; its leaves are elliptic-oblanceolate to oblong-elliptic; and it bears terminal racemes with yellow flowers-each inflorescence producing more than 10 flowers-with labella marked by reddish-brown spots (Chen and Lang, 1986; Chen et al., 2013). These characteristics clearly distinguish it from other *Cypripedium* species. Geographically, *C. subtropicum* is mainly distributed in China, including Motuo County (Tibet Autonomous Region); Funing, Malipo, Xichou, Maguan, Pingbian, Longling, Tengchong, and Gongshan counties (Yunnan Province); and Napo and Tianlin counties (Guangxi Zhuang Autonomous Region). It also occurs in northern Vietnam (Jiang and Liu, 2009). Despite its relatively broad distribution range, *C. subtropicum* occupies extremely narrow suitable habitats, and each discovered population is extremely small, qualifying it as a typical species with a critically small population. Owing to its multi-flowered trait and unique labellum, *C. subtropicum* holds exceptionally high ornamental value, which has triggered excessive wild collection. Combined with habitat destruction and population degradation, the number of wild individuals has declined annually, pushing the species to the brink of extinction. Although *C. subtropicum* was once recorded in multiple regions across China, over-collection driven by its ornamental, economic, and research value has led to its extinction in many former distribution areas (e.g., Pingbian, Funing, and Tengchong counties). Currently, fewer than 200 wild *C. subtropicum* individuals remain, and this population size continues to decrease gradually. *C. subtropicum* is listed in Appendix I of the Convention on International Trade in Endangered Species of Wild Fauna and Flora (CITES) and classified as Endangered (EN) in the IUCN Red List of Threatened Species (Rankou and Averyanov, 2014; Hua et al., 2023). To enhance the conservation of *C. subtropicum*, Chinese forestry authorities first included this species in the National Conservation Program for Wild Plants with Extremely Small Populations (2011–2015).

Orchidaceae plants have attracted global attention from countries and conservation organizations, and are recognized as a key group in biodiversity conservation. *C. subtropicum* stands as one of the flagship species for Orchidaceae conservation, with unique ecological and evolutionary significance: it is not only the sole subtropical *Cypripedium* species in Asia, but also the extant representative with the closest phylogenetic relationship to the tropical American genus *Selenipedium* within the Cypripedioideae (Chen and Lang, 1986). Consequently, research on *C. subtropicum* carries significant scientific value. Molecular phylogenetic studies have revealed highly conserved nucleotide sites in the ITS region between *C. subtropicum* and *Selenipedium* (e.g., adenine at position 93, guanine at positions 123 and 603), supporting its status as a relatively primitive lineage within *Cypripedium* (Li et al., 2011). Geographically, *C. subtropicum* presents a disjunct distribution and shows intercontinental floristic correspondence with the genus *Selenipedium* in Central America, implying that both taxa may have evolved from a relict lineage of Tertiary paleotropical flora. Furthermore, ecophysiological and reproductive biology investigations have further clarified the adaptive characteristics and endangered mechanisms of *C. subtropicum*. As an evergreen species retaining leaves in winter, it exhibits traits adapted to shaded understory environments, including low leaf thickness (LT), high chlorophyll concentration, and low light saturation point (LSP) and light-saturated photosynthetic rate. however, its narrow light adaptation range and low dark respiration rate limit competitiveness in strong light or excessive shading-key physiological factors contributing to its endangered status (Jiang et al., 2024; Zhang et al., 2021). To date, systematic investigations on *C. subtropicum* have covered fields such as pollination ecology (Jiang et al., 2020), chloroplast genomics (Li et al., 2011; Guo et al., 2021), and seed propagation (Perner et al., 2022; Hua et al., 2023).

Root-associated fungal communities (root-endosphere and rhizosphere soil) are critical for orchid growth, nutrient acquisition, stress resistance, and habitat adaptation, participating in key life cycle processes that underpin the conservation of endangered species (Swarts and Dixon, 2009; Rasmussen et al., 2015; Luo et al., 2020, 2020; Zhao et al., 2021; Li et al., 2021a, 2021; Liang et al., 2022). In addition to mycorrhizal fungi that have direct effects on seed germination, protocorm and plant growth of Orchidaceae plants, root-associated fungi also exert a significant impact on the life cycle of Orchidaceae plants (Liu et al., 2010; Li et al., 2022). Conservation measures such as *ex situ* reintroduction are closely related to the activities of root-associated fungi. Numerous studies have explored the fungal communities of other *Cypripedium* species using Illumina MiSeq high-throughput sequencing and pure culture techniques (Sim et al., 2010; Lindahl et al., 2013; Lee et al., 2015). Gang et al. (2017) investigated the rhizosphere and endophytic fungal communities of *C. japonicum* in its native habitats. They isolated a total of 519 fungal strains, which were classified into 65 genera and 119 species. Notably, *Trichoderma* was the dominant genus in both compartments, and endophytic fungi showed higher diversity despite the similar overall community structure between rhizosphere and endophytic samples (Gang et al., 2017). Cho et al. (2022) found that *Lycoperdon nigrescens* contributed to the natural habitat expansion of *C. japonicum* and *Russula violeipes* served as an indicator taxon for its successful *ex situ* cultivation. These findings offer critical insights for the restoration of endangered orchid populations in natural habitats (Cho et al., 2022). Fu et al. (2019) compared the fungal communities in rhizosphere and non-rhizosphere soils of *C. macranthos*, finding that rhizosphere soils had higher fungal richness and diversity, with *Mycena* and *Ceratobasidium* as dominant taxa. They obtained 1223 OTUs in total, with rhizosphere soils containing more OTUs than non-rhizosphere soils and 726 shared OTUs between the two (Fu et al., 2019). Fan et al. (2024) found significant differences in endophytic fungal abundance between *C. macranthos* roots and rhizosphere soils (with higher loads in rhizosphere soils), no shared OTUs or genera between the two, and higher fungal richness but non-significant diversity differences in rhizosphere soils. They also observed strong compartment-specificity in fungal distributions, with Rozellomycota having uniform relative abundance, *Malassezia* exclusive to roots, and *Sebacina* restricted to rhizosphere soils. Zhao et al. (2014) obtained 8 mycorrhizal fungal ITS sequences from wild *C. yunnanense* and *C. guttatum* in northwestern Yunnan using rDNA ITS amplification and MEGA-based analysis (Zhao et al., 2014). Miao et al. (2015) identified 388 monoclonal sequences from five endangered *Cypripedium* species in the Shangri-La region, which clustered into 8 genera, demonstrating high fungal richness and diversity in these taxa (Miao et al., 2015). Weerasuriya et al. (2022) recovered a broad range of fungi from *C. reginae* roots, including arbuscular mycorrhizal fungi, ectomycorrhizal fungi, a generalist endophyte, and pathogens (Weerasuriya et al., 2022). Moreno-Camarena and Ortega-Larrocea (2022) emphasized that mycorrhizal isolates determine orchid seed compatibility and post-germination development, noting that fungi from adult *C. irapeanum* belong to two distinct families, implying potential host-specificity and fungal restriction in related *Cypripedium* species (Moreno-Camarena and Ortega-Larrocea, 2022). Xu et al. (2019) examined three *Cypripedium* species along an elevation gradient in Huanglonggou, Sichuan, and found that 99.6% of the detected OTUs belonged to *Tulasnellaceae*. They also found that elevation reduced the mycorrhizal diversity of *C. tibeticum* but had no effect on *C. flavum* or *C. bardolphianum*, with significant differences in community structure among the three *Cypripedium* species, indicating strong host preference of *Cypripedium* mycorrhizal fungi (Xu et al., 2019). However, research on diversity and community structure of root-associated fungal communities of *C. subtropicum* remains notably scarce, creating a critical knowledge gap for its conservation. Therefore, this study aimed to systematically analyze similarities and differences of fungal communities in root-endosphere and rhizosphere soil samples of *C. subtropicum* across different regions and populations. Illumina Novaseq high-throughput sequencing, ASV, composition analysis, Alpha and Beta diversity and co-occurrence network analysis were used to characterize fungal communities structure and diversity of root-associated *C. subtropicum* in four Yunnan regions (Wenshan, Baoshan, Nujiang, Kunming) and three population types (wild, introduced, cultivated). The findings will provide a scientific basis for the subsequent research on the conservation and reintroduction of *C. subtropicum*.

## 2 Materials and methods

### 2.1 Plant material and soil sampling

**Figure 1:**
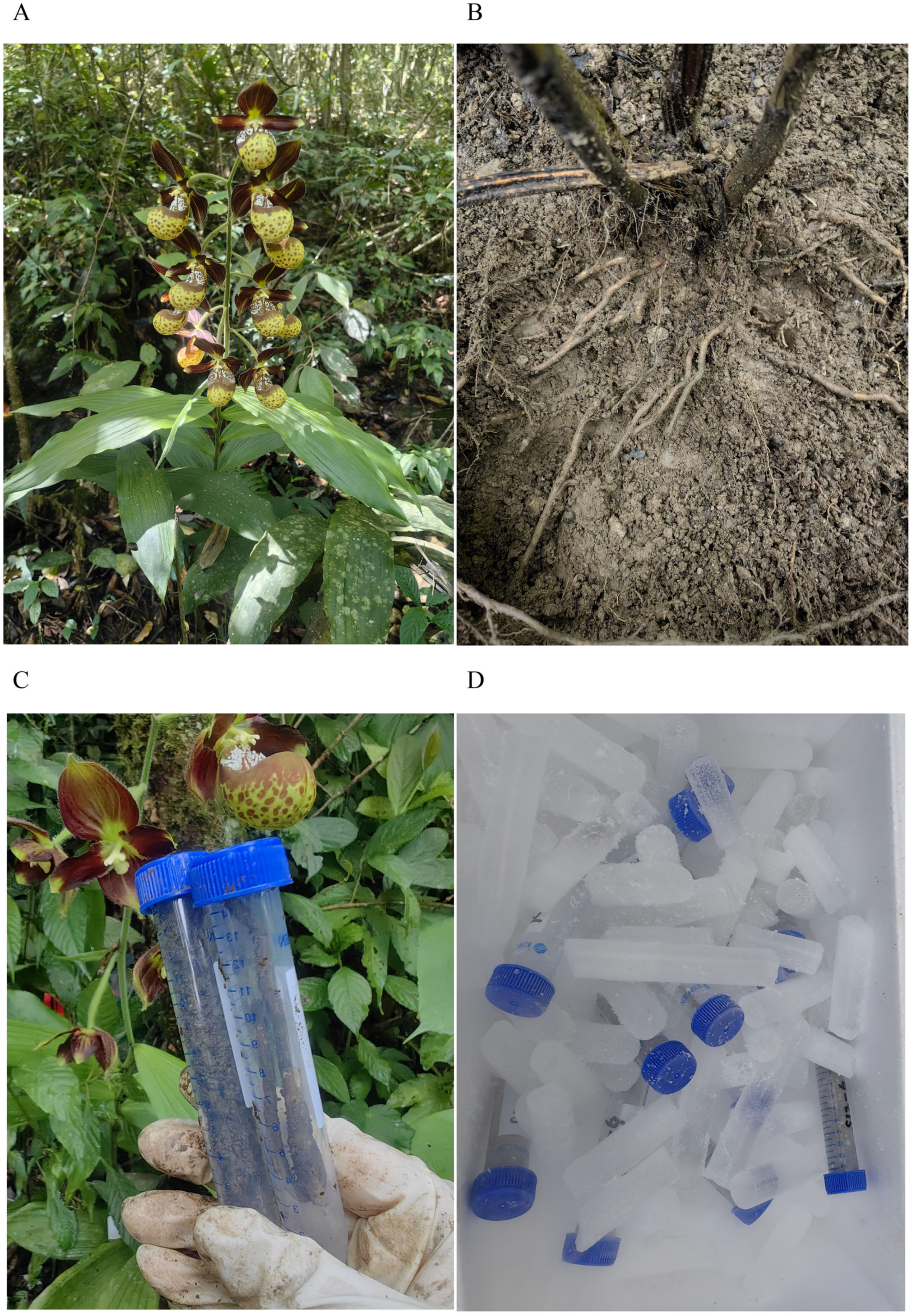
Samples of *C. subtropicum.* [**(A)** Wild growth status of *C. subtropicum*; **(B)** Roots and rhizosphere soil of *C. subtropicum*; **(C)** Collection of roots and rhizosphere soil samples; **(D)** Preservation of roots and rhizosphere soil samples].

In order to investigate the influence of regions on diversity and structure of root-associated fungal communities of *C. subtropicum*, the samples from different regions (Malipo county of Wenshan prefecture, Maguan county of Wenshan prefecture, Xichou county of Wenshan prefecture, Longling county of Baoshan prefecture, Gongshan county of Nujiang prefecture, and Kunming) in Yunnan province were also collected, which included three life forms of *C. subtropicum* (wild, introduced, and planted individuals), and covered most of the existing distribution sites of *C. subtropicum* (**Figure 1**). The population situation and details of *C. subtropicum* samples are shown in **Table 1**. From each sampling plots of every population, five clumps of *C. subtropicum* were randomly selected, and current-year roots, together with rhizosphere soil samples, were collected for high-throughput sequencing. For rhizosphere soil sampling, the core collection scope was strictly defined as the soil within 0–2 mm of the root surface (i.e., the microdomain closely influenced by root exudates), which is the key range to reflect the interaction between the plant and soil microorganisms. During sampling, an ethanol-sterilized shovel was used to remove surface soil, and sterile forceps were employed to collect root segments (about 10 cm in length) and rhizosphere soil (about 5 g). All samples were placed into 15 mL sterile centrifuge tubes and transported with liquid nitrogen quick-freezing to maintain freshness. A total of 120 samples were collected from 12 population sampling plots (P1-P12), including 60 root samples and 60 rhizosphere soil samples. For each sample at every sampling plot, root samples and soil samples were sequentially numbered with the prefixes R and S, respectively. For instance, the sampling site of Maguan County in Wenshan was designated as plot 1 (P1); the first root sample was labeled R1.1, and its corresponding rhizosphere soil sample was marked S1.1. S1 includes 5 rhizosphere soil samples from sampling site P1, R1 includes 5 root-endosphere samples from sampling site P1. All remaining samples were numbered following the same rule in sequence. The soil types in the habitat of *C. subtropicum* include humus soil, clay soil, red soil and sandy soil, and all these soil types were covered in the rhizosphere soil sampling. For root preprocessing: the collected root segments were first rinsed sequentially with sterile phosphate-buffered saline (PBS) to completely remove residual rhizosphere soil adhering to the root surface. After thorough rinsing, the remaining surface-sterilized root tissues were regarded as the root-endosphere compartment, ensuring no contamination by rhizosphere soil microorganisms. All processed root and soil samples were stored at -80°C until microbial DNA extraction.

**Table 1:** Habitats and population profile of *C. subtropicum*.

Plot	Location	Altitude (m)	Slope (°)	Number of plants (cluster)	Average plant height (cm)	Average number of aerial stems	Population status
P1	Maguan county Wenshan, Yunnan	1504	40	6	100-120	4	Wild
P2	Luoshuidong, Malipo county, Wenshan, Yunnan	1458	45	93	90-120	4	Wild
P3	Orchid nursery, Malipo county, Wenshan, Yunnan	1196	40	20	70-80	3	Introduced from Gulinqing, Maguan county (2018)
P4	Orchid nursery, Malipo county, Wenshan, Yunnan	1200	35	30	90-120	4	Introduced from Vietnam (2018)
P5	Orchid nursery, Malipo county, Wenshan, Yunnan	1157	30	30	60-70	3	Introduced from Vietnam (2022)
P6	Taihetang, Malipo county, Wenshan, Yunnan	1481	30	30	90-110	3	Introduced from Xiajinchang, Malipo county (2009)
P7	Daxiechang, Malipo county, Wenshan, Yunnan	1462	25	30	60-70	2	Introduced from Vietnam (2018)
P8	Xichou county, Wenshan, Yunnan	1513	35	25	50-80	3	Wild
P9	Wangou,Malipo county, Wenshan, Yunnan	1432	30	7	20-30	2	Wild
P10	Longling county, Baoshan, Yunnan	1355	25	5	15-20	3	Wild
P11	Gongshan county, Nujiang, Yunnan	1468	30	8	80-120	5	Wild
P12	Yunnan Academy of Forestry and Grassland, Kunming, Yunnan	1935	–	35	10-15	3	The seedlings were planted in 2021

### 2.2 DNA extraction and sequencing

ALFA Soil DNA Extraction Kit (Guangzhou Findrop Biotechnology Co., Ltd., China) was used to extract total microbiome DNA from all samples, the purity and concentration of the DNA were assessed using a Nanodrop One (Thermo Fisher Scientific, MA, USA). Using genomic DNA as the template, PCR amplification was performed according to the selected sequencing regions, employing specific primers bearing barcodes and Premix Taq (EX Taq Version 2.0 plus dye,Takara Biotechnology, Dalian Co. Ltd., China). Specific primers with barcode were synthesized according to the designated sequencing area. PCR amplification primers corresponding to the region of ITS1F_ITS2R (Blaalid et al., 2013; Lindahl et al., 2013; Lemons et al., 2017) the upstream primer ITS1F: CTTGGTCATTTAGAGGAAGTAA, and the downstream primer ITS2R: GCTGCGTTCTTCATCGATGC. PCR amplification was performed using Analytikjena Biometro TONE 96G. PCR reaction system includes: 25 μL 2x Premix Taq, 2 μL upstream primer (10 μM), 2 μL downstream primer (10 μM), DNA 50 ng, supply nuclease-free water to 50 μL. PCR reaction conditions: 1) 95°C 3 min; 2) 32 cycle of 95°C 20 s, 56°C 30 s,72°C 30 s; 3) 72°C 5 min; 4)12°C Hold. Electrophoresis of PCR products was conducted on a 1.5% agarose gel to assess the fragment length and concentration. Samples with main bands falling within the normal range were suitable for further experimentation. Following concentration comparison of PCR products using GeneTools Analysis Software (Version 4.03.05.0, SynGene), the required volume for each sample was calculated based on the principle of equal mass, after which the PCR products were mixed. The PCR mixture was then recovered using the E.Z.N.A.^®^ Gel Extraction Kit (Omega, USA) D2500-03, with target DNA fragments eluted in TE buffer. Library construction was carried out following the standard protocol of the ALFA-SEQ DNA Library Prep Kit, and the size of the library fragments was evaluated on the Qsep400 High-Throughput Nucleic Acid & Protein Analysis System (Hangzhou Houze Biotechnology Co., Ltd., China). The concentration of the library was measured using a Qubit4.0 (Thermo Fisher Scientific, Waltham, USA). The constructed amplicon libraries were subjected to PE250 sequencing on the Illumina Novaseq 6000 platform. (Guangdong Magigene Biotechnology Co., Ltd., Guangzhou, China).

## 3 Data analysis

### 3.1 Sequencing data processing

Paired-end Raw Reads Data Filtering: FASTQ preprocessing software (fastp, an ultra-fast all-in-one FASTQ preprocessor, version 0.14.1, https://github.com/OpenGene/fastp) was utilized to separately perform sliding window quality trimming on raw reads from both ends of paired-end data with the parameters: -W 4 -M 20 (for quality control). According to the official function of fastp, -W specifies the sliding window size (4 bp) and -M denotes the minimum mean quality score (20, equivalent to Q 20) required for the window to be retained, which is consistent with the sliding window quality trimming function of fastp. Concurrently, cutadapt software (version 1.14, https://github.com/marcelm/cutadapt/) was used to remove primers from the data based on the primer information at the 5’ and 3’ ends of sequences with the parameter: -e 0.2 (maximum allowed error rate for primer matching), thus obtaining quality-controlled paired-end Clean Reads.

Paired-end Clean Reads Assembly: For paired-end sequencing data, relying on the overlap relationship between PE reads, the -fastq_mergepairs command of usearch software (version 10.0.240, http://www.drive5.com/usearch/) was employed with default parameters for AVS assembly. The preset parameters (minimum overlap length of 16 bp and maximum 5 bp mismatches in the overlapping region) are consistent with the basic configuration of usearch -fastq_mergepairs for read assembly, which is used to exclude non-conforming Tags and generate Raw Tags.

Raw Tags Sequence Quality Filtering: fastp software (an ultra-fast all-in-one FASTQ preprocessor, version 0.14.1, https://github.com/OpenGene/fastp) was used again to perform sliding window quality trimming on the Raw Tags data with the parameters: -W 4 -M 20 (for quality control, consistent with the parameters used in raw reads filtering). This operation is in line with the quality control function of fastp, ultimately yielding valid assembled segments, referred to as Clean Tags.

OTU Denoising: QIIME dada2 (QIIME2, version v2020.11.0) was used for OTU denoising with the parameter: --p-trunc-len 0 (no truncation of reads, retaining the full length of Clean Tags for subsequent analysis), which is a standard parameter setting for QIIME dada2 when full-length read retention is required.

Species Annotation: The -sintax command of usearch software (version 10.0.240, http://www.drive5.com/usearch/) was utilized for species annotation with the default threshold of 0.8 (minimum confidence threshold for assigning taxonomic classification to ASVs), which is consistent with the default confidence threshold setting of usearch-sintax for species annotation

### 3.2 ASV analysis

In order to facilitate the analysis of the species and number of fungi in the sequencing results, divisive amplicon denoising algorithm 2 (DADA2:high-resolution sample inference from Illumina amplicon data) was used to generate amplicon sequence variants (ASVs) by denoising of the demultiplexed sequencing reads (Callahan et al., 2016). After denoising, Amplicon sequence variants (ASVs) were obtained by inferring true sequence variations through an error model. Utilize qiime feature-classifier classify-sklearn to align the representative sequence of each ASV against Unite 8.0 (ITS) databases for species-level annotation, with a default confidence threshold of 0.8 and default databases being Unite 8.0 (ITS) (Nilsson et al., 2019). Based on the minimum number of sequencing reads among all samples, rarefaction curve rarefaction analysis was performed according to the minimum sample size. This process aims to determine the taxonomic origin of all sequences. The taxonomy results are classified into seven hierarchical levels: kingdom (L1), phylum (L2), class (L3), order (L4), family (L5), genus (L6), and species (L7). Exclude representative sequence annotated as chloroplasts or mitochondria (for 16S amplicons) and those failing to be assigned to the kingdom level, and remove all unannotated representative sequences across the taxonomic ranks of kingdom, phylum, class, order, family, genus, and species, resulting in the final set of valid Tag sequences (No. of seqs) and the comprehensive representative sequence taxonomy table (ASV_table) for each sample in the subsequent analyses. Based on the ASV_table obtained after eliminating singleton ASV, chimeras, and contaminating representative sequence as described above, calculate the number of reads and ASVs present in individual samples or groups. All subsequent analyses were performed on the Magigene Cloud Platform, with the website: http://tools.magigene.com/h5-BioCloud-console/report/14_A17A03?projectId=ff8080819918042a01992832c584000f&taskId=ff8080819a3f3500019a4cab1ba41c63&appId=14&taskVersion=1.

### 3.3 Species community structure

Perform shared and unique species enumeration, community composition analysis, and species abundance clustering analysis using R software. Through the community Bar diagram to analyze the fungal community at all levels of classification groups and dominant species. The occurrence of different taxonomic ranks across samples and groups was counted to construct Venn diagrams. In the diagrams, different colors represent different samples/groups; overlapping regions indicate the number of shared taxonomic ranks among samples/groups, while non-overlapping regions represent the number of unique taxonomic ranks specific to each sample/group. Venn diagrams can be used to display the number of shared and unique AVSs across multiple samples, intuitively illustrating the AVS overlap among samples. The bar chart of microbial community composition was constructed by summarizing sequence information at different taxonomic ranks (including AVS-related taxonomic levels) from the AVS table, calculating the relative abundance of each taxon. Meanwhile, only taxa with a relative abundance of more than 0.01% were selected, and the top 10 taxa with the highest abundance were used for plotting. During the plotting process, unannotated taxa and other miscellaneous taxa were excluded. The species abundance heatmap was used to represent the community composition and abundance of one or more samples at a specific taxonomic level.

### 3.4 Community composition cluster analysis

To explore the similarity and difference of the fungal communities composition among all root-associated samples of *C. subtropicum*, community composition cluster analysis were constructed at genus and species levels. For each level, only the top 10 taxa ranked by overall relative abundance were selected for cluster construction. The visualization of these cluster was integrated with two key datasets: the relative abundance of each taxon across all samples, and the confidence scores of species-level taxonomic annotations, to provide a comprehensive insight into fungal community structure and composition cluster. The integrated composition cluster visualization adopted a dual-panel layout. The left panel displays the cluster tree topology, which was generated using the unweighted pair-group method with arithmetic mean (UPGMA) algorithm; a shorter branch length between two samples in this topology indicates a higher similarity in their fungal community composition. The right panel presents the distribution of taxon-specific relative abundances across all samples for the corresponding taxonomic level, enabling direct linkage between composition cluster and community abundance profiles.

### 3.5 Alpha diversity analysis

Alpha diversity was used to explore the diversity of microbial communities within the sample and the richness and evenness between communities by means of a variety of statistical indices (Baczkowski et al., 1998). Based on the ASV abundance table, compute diversity indices (richness, chao1, shannon_e, simpson, goods_coverage), using usearch -alpha_div (version V10, http://www.drive5.com/usearch/) and R package. Based on the ASV abundance table, perform rarefaction curve calculations for the diversity indices using usearch -alpha_div_rare (version V10, http://www.drive5.com/usearch/) and R package. Perform inter-group difference analysis for alpha diversity indices using R software. This analysis will separately employ parametric and non-parametric tests. Alpha diversity characterizes species diversity within sample (i.e., within-habitat diversity), independent of other samples, and is primarily quantified by species richness and evenness. Boxplots were employed to visually present the median, dispersion degree, maximum value, minimum value, and outliers of alpha diversity indices across groups. To verify the significance of intergroup differences, Kruskal-Wallis rank sum test were conducted. Four core alpha diversity indices (Chao1 (species richness estimator), Shannon-Wiener (Shannon-E, comprehensive diversity), Simpson (dominance index), and Good’s coverage (sequencing completeness)) were selected for analysis across different regions, habitats, and population types of *C. subtropicum*. Chao1 index quantifies community species richness by extrapolating the total number of extant species based on the distribution of rare taxa (i.e., those detected once or twice). It is positively correlated with species richness, where higher values indicate greater actual species number and community diversity. Shannon-E index, a comprehensive metric integrating both species richness and abundance evenness, which quantifies community diversity via the uncertainty of species composition. Higher values correspond to richer species and more uniform abundance distribution, representing greater community diversity. Simpson index reflects community dominance and is inversely related to diversity. Lower values signify higher species diversity, while higher values indicate the dominance of a few taxa and lower overall diversity.

### 3.6 Beta-diversity analysis

Based on the ASV abundance table, employ the ‘vegan’ package in R software to perform Nonmetric Multidimensional Scaling (NMDS) analysis utilizing bray-curtis distance algorithms. which was employed as the core method for beta diversity analysis to assess variations in fungal community composition across different geographic regions. NMDS is a multivariate ordination technique based on sample distance matrices, which conducts rank-ordering of pairwise sample distances to project samples into a low-dimensional ordination space. Its core objective is to maximize the consistency between the ordination distances of samples in the reduced-dimensional space and their original pairwise distance relationships, thereby enabling intuitive visualization of community compositional differences populations. Notably, NMDS is unaffected by the absolute values of sample distance metrics, and only considers their relative magnitudes, making it robust to data heterogeneities. As a nonlinear ordination model, it generates more stable and reliable ordination results for complex datasets with high dimensionality and heterogeneous taxon distributions. The significance analysis of inter-group community structure differences refers to calculating the distance difference matrix based on the compositional composition of each sample at the ASV (amplicon sequence variant) classification level using the Bray-Curtis distance algorithm, and then verifying the significance of inter-group community structure differences by means of Adonis analysis.

### 3.7 Co-occurrence network analysis

Based on the ASV abundance table, R software was used to perform co-linearity network analysis on the species abundance information across different samples. Co-occurrence network analysis is a powerful tool for visualizing distribution patterns and interspecific associations among microbial taxa across environmental samples, as well as for revealing compositional similarities and differences between samples. This approach involves conducting correlation analyses on species abundance datasets from distinct samples to quantify coexistence relationships of target taxa across heterogeneous environments (Pearson or Spearman correlation). The network is clustered based on the number of shared taxa among samples, where a higher number of shared species indicates closer compositional relatedness between the corresponding samples.

## 4 Results and analysis

### 4.1 Number of valid tag sequences and ASVs in root-associated *C. subtropicum*

A total of 120 root-associated *C. subtropicum* samples were collected, including 60 root-endosphere samples and 60 rhizosphere soil samples. Sampling sites covered six locations in Yunnan Province: Malipo County, Maguan County, and Xichou County (Wenshan Zhuang and Miao Autonomous Prefecture); Longling County (Baoshan Prefecture); Gongshan County (Nujiang Lisu Autonomous Prefecture); and the Yunnan Academy of Forestry and Grassland (Kunming), and including wild, introduced, and cultivated populations of three growth types (**Table 1**). The flowering status, root growth characteristics, and sampling conditions of *C. subtropicum* roots and rhizosphere soils are illustrated in **Figure 1**. Given that habitat type, geographic region, and altitude are known to influence the diversity and composition of root-associated fungal communities in orchid roots and rhizosphere soils, these factors were considered as key variables in subsequent analyses. After DNA extraction, Illumina Novaseq high-throughput sequencing, paired-end read quality control and assembly, a total of 10,098,679 clean high-quality sequences were obtained. Among these, 4,988,933 sequences were derived from root-endosphere samples and 5,109,746 from rhizosphere soil samples, with root-endosphere sequences accounting for 97.64% of rhizosphere soil sequences (**Supplementary Table 1**). After all samples were denoised, subjected to rarefaction analysis according to the minimum sample sequence number, and the mitochondria, chloroplasts and unannotated taxa were removed, a total of 2,166,708 valid tag sequences and 39,386 ASVs were obtained. 1,111,782 valid tag sequences and 19,661 ASVs were from root-endosphere samples, and 1,054,926 valid tag sequences and 19,725 ASVs were from rhizosphere soil samples. The difference in the number of ASVs between the two sample types is only 0.33%.

Root-associated samples from Wenshan Prefecture (Maguan, Malipo, and Xichou counties) contributed 34,564 ASVs (combined from roots-endosphere and rhizosphere soils samples), accounting for 87.76% of the total ASVs across all regions, with an average of 384 ASVs per sample. In contrast, root-associated samples from Baoshan Prefecture (Longling County) yielded 2,125 total ASVs (average: 213 ASVs per sample). Root-associated samples of Nujiang Prefecture (Gongshan County) were obtained 1,794 total ASVs (average: 179ASVs per sample), and root-associated samples of Kunming (the Yunnan Academy of Forestry and Grassland) were obtained only 903 total ASVs (average: 90 ASVs per sample; **Supplementary Table 2**). The numbers of ASVs in root-associated samples of *C. subtropicum* differed significantly among different geographic regions.In terms of the average number of ASVs, the root-associated samples from Wenshan Prefecture exhibited the highest value, which was more than four times that of the samples from Kunming. Baoshan samples ranked second, followed by Nujiang samples. These results confirm that *C. subtropicum* growing in Wenshan Prefecture exhibit the highest fungal abundance in root-endosphere and rhizosphere soil, followed by those in Baoshan and Nujiang, while Kunming populations exhibit the lowest fungal richness.

**Figure 2:**
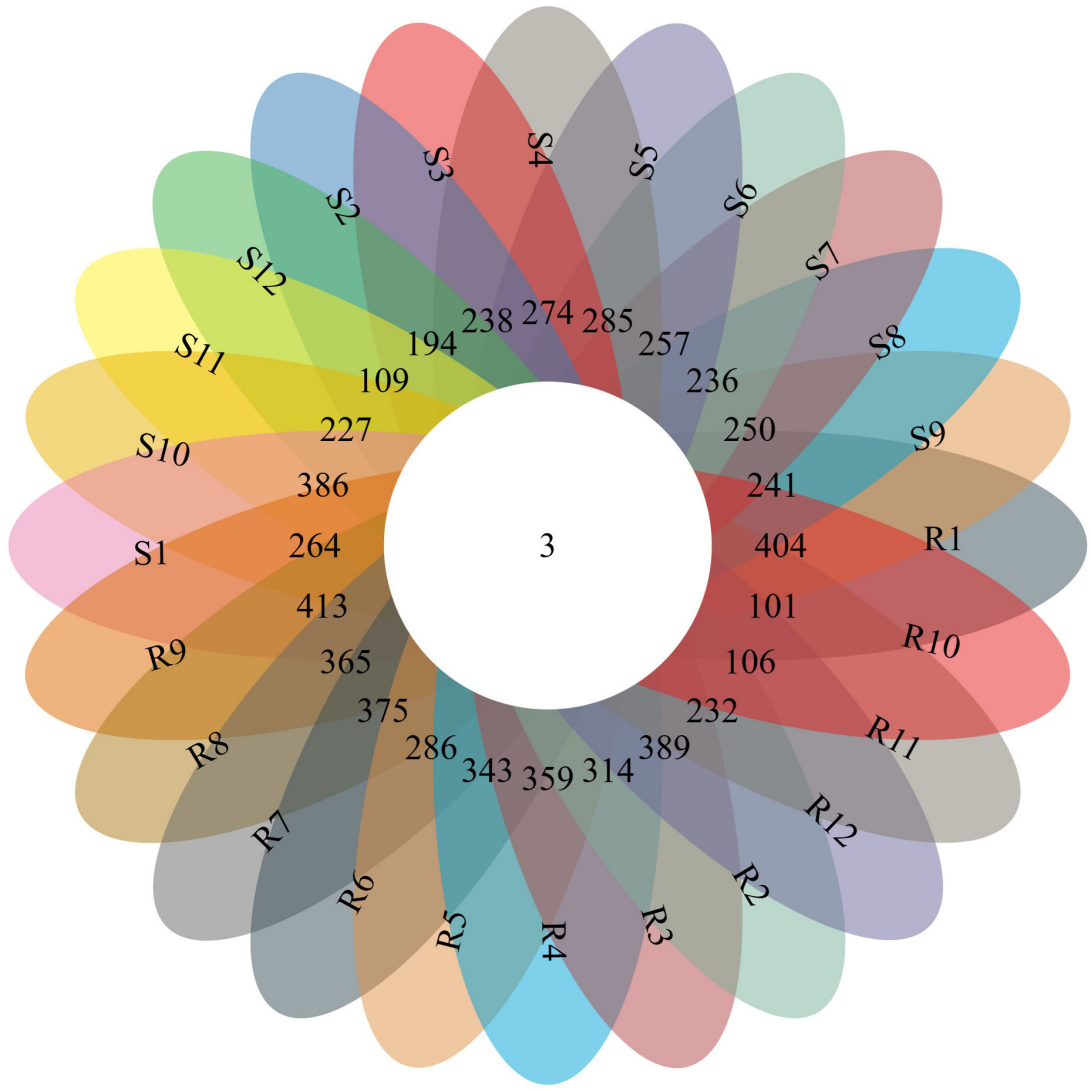
Venn chart of root-endosphere (R1–12) and rhizosphere soil (S1-12) samples across all 120 samples from 12 habitats. Different colors represent different samples, the number of overlapping parts represents the number of species shared by multiple samples, and the number of non-overlapping parts represents the number of species unique to the corresponding sample.

Venn diagrams were used to visualize shared and unique fungal ASVs among samples, to assess the relationships between fungal communities in root-endosphere and rhizosphere soil samples across different regions, habitats and population types. Three fungal ASVs were shared between root-endosphere and rhizosphere soil samples across all 120 samples from 12 habitats (**Figure 2**). These core shared taxa were identified as follows: ASV 8: k: Fungi, p: Basidiomycota, c: Agaricomycetes, o: Sebacinales, f: Sebacinaceae, g: *Sebacina*, s: *Sebacina* sp., ASV 36: k: Fungi, p: Ascomycota, c: Eurotiomycetes, o: Chaetothyriales, f: Herpotrichiellaceae, g: *Exophiala*, s: *Exophiala equina*, ASV 47: k: Fungi, p: Ascomycota, c: Dothideomycetes, o: Dothideales, f: Aureobasidiaceae, g: *Aureobasidium*, s: *Aureobasidium* sp. Root-endosphere samples shared 5 ASVs across all 60 root samples from 12 habitats. In addition to the three core ASVs (ASV 8, ASV 36, ASV 47), the root-exclusive shared taxa were: ASV 39: k: Fungi, p: Ascomycota, c: Sordariomycetes, o: Hypocreales, f: Nectriaceae, g: *Fusarium*, s: *Fusarium oxysporum*, ASV 92: k: Fungi, p: Ascomycota, c: Sordariomycetes, o: Hypocreales, f: Nectriaceae, g: *Fusarium*, s: *Fusarium solani*. Rhizosphere soil samples shared 5 ASVs across all 60 rhizosphere samples from 12 habitats. Beyond the three core ASVs, the rhizosphere-exclusive shared taxa were: ASV_3: k: Fungi, p: Mortierellomycota, c: Mortierellomycetes, o: Mortierellales, f: Mortierellaceae, g: *Mortierella*, s: *Mortierella* sp., ASV 239: k: Fungi, p: Basidiomycota, c: Tremellomycetes, o: Filobasidiales, f: Piskurozymaceae, g: *Solicoccozyma*, s: *Solicoccozyma terricola*. Among the four study regions (Wenshan, Baoshan, Nujiang, Kunming), the root-associated samples of Wenshan Zhuang and Miao Autonomous Prefecture (P1-9) shared 6 ASVs. The shared taxa included ASV 3 (*Mortierella* sp.), ASV 62 (*Mortierella* sp.), ASV 8 (*Sebacina* sp.), ASV 14 (*Cordana bisbyi*), ASV 52 (*Fusarium solani*) and ASV 71 (*Metarhizium _marquandii*). The root-associated samples of Baoshan Prefecture (Longlin County, P10) shared 3ASVs, and were identified as ASV 10 (*Ilyonectria robusta*), ASV 298 (*Filobasidium magnum*) and ASV 1146 (*Rhodotorula mucilaginosa*). The root-associated samples of Nujiang Prefecture (Gongshan County, P11) shared only ASV 10 (*Ilyonectria robusta*) and ASV 250 (*Archaeorhizomyces* sp.). The root-associated samples of Kunming (cultivated population, P12) shared 5 ASVs, which were ASV_11 (*Byssochlamys_sp.*), ASV_47 (*Aureobasidium* sp.), ASV_68 (*Serendipita_sp*), ASV 139 (*Phialemonium inflatum*) and ASV 192 (*Alternaria* sp.). Within Wenshan Prefecture, 56 ASVs were shared in root-associated samples of Maguan County (P1), 30 were in Xichou County (P8), and 11 were in Malipo County (P2-7, P9). In Malipo County, which included both wild and introduced populations, 21 ASVs were shared in root-associated samples of wild populations (P2, P9) and 16 were shared in introduced populations (P3-7; **Supplementary Table 3**).

### 4.2 Composition analysis of root-associated fungal communities in *C. subtropicum*

Species abundance analysis is a robust approach to characterize the community composition and taxon abundance of multiple samples at a specified taxonomic level. Taxonomic analysis (excluding unannotated taxa) at three levels revealed that Ascomycota, Basidiomycota and Mortierellomycota were the most representative phyla in all root-associated sammples in 12 habitats of four sampling area. The top 10 dominant genera (by abundance) were *Mortierella*, *Archaeorhizomyces*, *Sebacina*, *Fusarium*, *Metarhizium*, *Ilyonectria*, *Inocybe*, *Exophiala*, *Trichoderma*, *Cordana*, *Cladophialophora*. The top 10 dominant species (by abundance) were *Mortierella* sp.*, Archaeorhizomyces* sp.*, Sebacina* sp., *Fusarium solani*, *Ilyonectria robusta*, *Cordana bisbyi*, *Inocybe* sp., *Byssochlamys* sp.*, Serendipita* sp., *Cladophialophora* sp. The relative abundance and composition of root-associated fungal communities vary among different populations. Meanwhile, Population types (wild, introduced, cultivated) may affect the community composition of root-associated fungi. In the sampling plots, Maguan (P1), Xichou (P8), Longlin(P10), and Gongshan (P11) samples represented wild populations. Kunming (P12) samples were seed-germinated cultivated populations, whose seeds were derived from artificial pollination of the wild populations of *C. subtropicum* in Malipo County. Malipo (P2-7, P9) samples included both wild and introduced populations.

**Figure 3:**
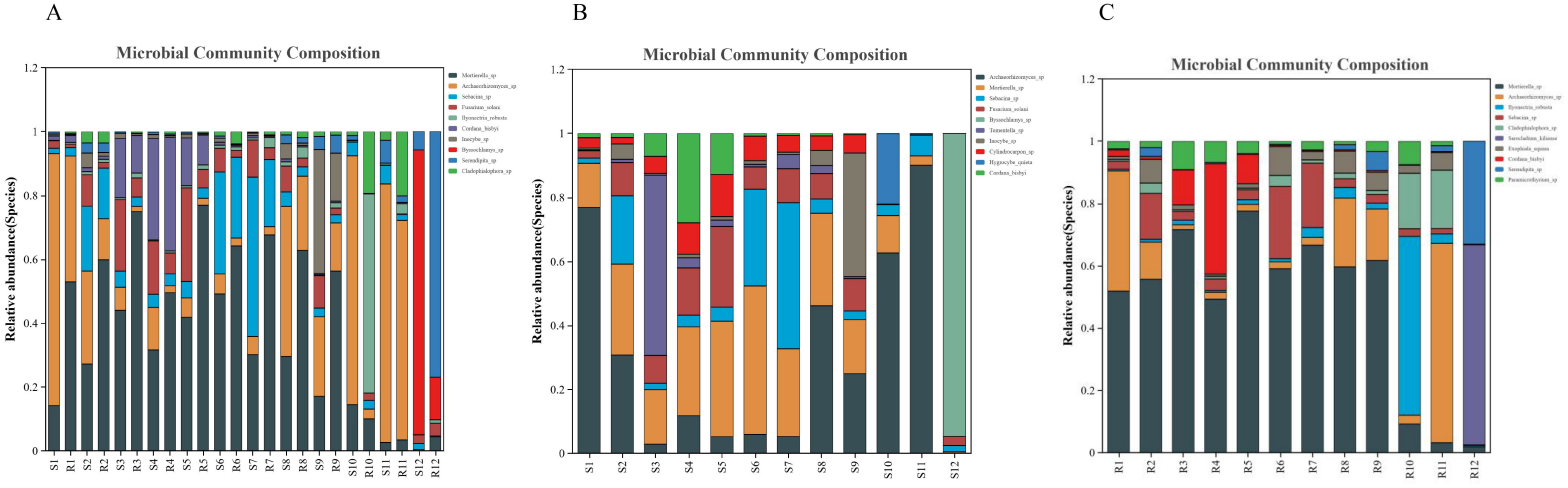
Bar diagram of mycorrhizal fungal community composition. The abscissa is the sample name, and the ordinate is the proportion of species in the sample. Columns with different colors represent different species, and the length of the column represents the proportion of the species. **(A)** root-endosphere and rhizosphere soil samples of 12 habitats at species level; **(B)** rhizosphere soil samples of 12 habitats at species level (S1-12); **(C)** root-endosphere samples of 12 habitats at species level (R1-12). The result of S1 is the mean value of 5 rhizosphere soil samples from sampling site P1, the result of R1 is the mean value of 5 root-endosphere samples from sampling site P1, and the results of the remaining samples are labeled in the same manner.

In 60 rhizosphere soil samples of 12 habitats, Ascomycota was the dominant phylum, and the relative abundance were from 40.3% to 78.0%. The relative high abundance of Ascomycota of rhizosphere soil samples came from Maguan (78.0%, P1) and Kunming (75.5%, P12). At the genus level, *Mortierella* (highest: 45.3% in Kunming, P12) and *Archaeorhizomyces* (highest: 84.6% in Gongshan, P11) were dominant. At the species level, *Archaeorhizomyces* sp. dominated in rhizosphere soil samples of wild populations (Maguan: 79.0%, P1; Longlin: 78.0%, P10; Gongshan: 81.0%, P11), while not be detected in Kunming cultivated samples, and *Byssochlamys* sp. (89.2%, P12) was exclusive to Kunming cultivated samples. *Mortierella* sp. was abundant in rhizosphere soil samples of *C. subtropicum* in Wenshan Area, the highest relative abundance reached 49.1% (P6), while was less abundant in other Areas, particularly in cultivated population (0.4%, P12). Ascomycota dominated all 60 root-endosphere samples (45.4%-75.7%), with the highest abundance in Gongshan (75.7%, P11). At the genus level, *Mortierella* dominated in Wenshan (42.5%-68.3%, P1-P9), while it was relatively low in abundance in root-endosphere samples in other areas. The dominated genus are respectively *Archaeorhizomyces* in Gongshan of Nujiang (57.2%, P11), *Ilyonectria* in Longlin of Baoshan (71.6%, P10), and *Fusarium* in Kunming (90.9%, P12). From the perspective of species taxonomic ranks, *Mortierella_sp* was particularly abundant in root-endosphere samples of *C. subtropicum* in Wenshan Area (49.5%-76.9%, P1-P9). *Archaeorhizomyces* sp.*, Ilyonectria_robusta* and *Serendipita* sp. were main fungi in root-endosphere samples of *C. subtropicum* in Gongshan of Nujiang (68.8%, P11), Longlin of Baoshan (62.5%, P10) and Kunming (77.0%, P12) (**Table 2**). The bar chart intuitively shows the composition and relative abundance of the root-associated fungal communities in 12 plots at the species taxonomic level (**Figure 3**). Across all 12 habitats, Ascomycota was the dominant phylum in both root-endosphere and rhizosphere soil samples, and *Mortierella*, *Archaeorhizomyces* were the dominant genera. The dominant species in the root-endosphere and rhizosphere soil samlples showed even greater regional variability.

**Table 2:** The relative abundance of fungal in root-endosphere and rhizosphere soil samples of *C. subtropicum* in 12 habitats (phylum, genus, species).

Phylum	S1	R1	S2	R2	S3	R3	S4	R4	S5	R5	S6	R6	S7	R7	S8	R8	S9	R9	S10	R10	S11	R11	S12	R12
Ascomycota	0.780	0.624	0.579	0.454	0.538	0.638	0.699	0.715	0.675	0.581	0.529	0.463	0.403	0.473	0.632	0.547	0.514	0.502	0.552	0.749	0.684	0.757	0.755	0.581
Basidiomycota	0.136	0.110	0.286	0.258	0.375	0.160	0.171	0.111	0.214	0.131	0.251	0.214	0.465	0.239	0.249	0.144	0.398	0.202	0.359	0.206	0.298	0.182	0.220	0.410
Mortierellomycota	0.084	0.266	0.135	0.288	0.087	0.202	0.130	0.174	0.111	0.288	0.220	0.323	0.131	0.288	0.119	0.309	0.088	0.296	0.089	0.045	0.018	0.061	0.024	0.008
Genus	S1	R1	S2	R2	S3	3	S4	R4	S5	R5	S6	R6	S7	R7	S8	R8	S9	R9	S10	R10	S11	R11	S12	R12
*Mortierella*	0.136	0.482	0.260	0.540	0.345	0.631	0.276	0.425	0.317	0.683	0.398	0.523	0.207	0.521	0.237	0.532	0.154	0.529	0.147	0.072	0.030	0.103	0.453	0.061
*Archaeorhizomyces*	0.723	0.346	0.254	0.110	0.050	0.013	0.104	0.018	0.044	0.019	0.049	0.019	0.039	0.019	0.367	0.195	0.218	0.139	0.768	0.021	0.846	0.572	0.000	0.000
*Sebacina*	0.015	0.023	0.177	0.137	0.035	0.024	0.032	0.030	0.037	0.028	0.247	0.200	0.334	0.158	0.038	0.025	0.024	0.023	0.042	0.019	0.061	0.015	0.162	0.003
*Fusarium*	0.029	0.015	0.093	0.022	0.223	0.074	0.189	0.081	0.267	0.063	0.085	0.027	0.083	0.033	0.083	0.024	0.099	0.023	0.001	0.016	0.001	0.025	0.375	0.909
*Metarhizium*	0.067	0.079	0.057	0.046	0.172	0.074	0.116	0.082	0.160	0.068	0.118	0.091	0.078	0.101	0.132	0.078	0.104	0.052	0.005	0.000	0.002	0.009	0.000	0.000
*Ilyonectria*	0.002	0.016	0.021	0.020	0.010	0.039	0.005	0.008	0.009	0.018	0.011	0.014	0.007	0.053	0.014	0.050	0.007	0.023	0.018	0.716	0.010	0.032	0.003	0.012
*Inocybe*	0.008	0.004	0.049	0.011	0.021	0.006	0.018	0.006	0.019	0.004	0.016	0.002	0.227	0.062	0.045	0.007	0.344	0.139	0.000	0.000	0.000	0.000	0.000	0.000
*Exophiala*	0.005	0.011	0.047	0.073	0.013	0.035	0.009	0.048	0.036	0.031	0.057	0.082	0.019	0.038	0.070	0.068	0.034	0.055	0.005	0.020	0.009	0.050	0.005	0.015
*Cordana*	0.013	0.019	0.011	0.009	0.129	0.096	0.248	0.293	0.108	0.081	0.007	0.004	0.005	0.003	0.007	0.004	0.003	0.004	0.001	0.000	0.000	0.003	0.000	0.000
*Cladophialophora*	0.001	0.005	0.031	0.033	0.002	0.009	0.002	0.008	0.001	0.005	0.013	0.038	0.002	0.012	0.008	0.016	0.013	0.012	0.014	0.135	0.040	0.191	0.002	0.000
Species	S1	R1	S2	R2	S3	R3	S4	R4	S5	R5	S6	R6	S7	R7	S8	R8	S9	R9	S10	R10	S11	R11	S12	R12
*Mortierella* sp.	0.141	0.529	0.271	0.599	0.440	0.749	0.315	0.495	0.418	0.769	0.491	0.642	0.300	0.677	0.295	0.627	0.170	0.563	0.145	0.100	0.026	0.034	0.004	0.044
*Archaeorhizomyces* sp.	0.790	0.394	0.292	0.128	0.072	0.016	0.134	0.022	0.061	0.022	0.063	0.024	0.058	0.026	0.471	0.232	0.250	0.151	0.780	0.031	0.810	0.688	0.000	0.000
*Sebacina* sp.	0.017	0.027	0.203	0.159	0.051	0.029	0.041	0.037	0.051	0.032	0.319	0.253	0.499	0.210	0.046	0.030	0.027	0.026	0.043	0.027	0.058	0.018	0.019	0.003
*Fusarium solani*	0.024	0.011	0.099	0.018	0.224	0.060	0.167	0.065	0.293	0.058	0.074	0.022	0.116	0.037	0.081	0.027	0.101	0.021	0.001	0.023	0.001	0.002	0.027	0.040
*Ilyonectria robusta*	0.001	0.006	0.010	0.010	0.006	0.016	0.003	0.006	0.007	0.014	0.009	0.011	0.007	0.032	0.014	0.035	0.003	0.017	0.004	0.625	0.006	0.032	0.000	0.011
*Cordana bisbyi*	0.014	0.021	0.013	0.010	0.186	0.118	0.317	0.356	0.149	0.093	0.010	0.005	0.007	0.004	0.009	0.004	0.004	0.005	0.001	0.000	0.000	0.004	0.000	0.000
*Inocybe* sp.	0.006	0.003	0.046	0.011	0.016	0.006	0.012	0.005	0.012	0.004	0.012	0.002	0.008	0.003	0.048	0.008	0.388	0.151	0.000	0.000	0.000	0.000	0.000	0.000
*Byssochlamys* sp.	0.000	0.000	0.000	0.000	0.000	0.000	0.000	0.000	0.000	0.000	0.000	0.000	0.000	0.000	0.000	0.000	0.000	0.000	0.000	0.000	0.000	0.000	0.892	0.133
*Serendipita* sp.	0.007	0.004	0.032	0.030	0.005	0.001	0.008	0.004	0.006	0.003	0.007	0.003	0.002	0.001	0.028	0.017	0.041	0.056	0.015	0.002	0.071	0.021	0.058	0.770
*Cladophialophora* sp.	0.001	0.006	0.035	0.036	0.001	0.005	0.002	0.008	0.002	0.005	0.014	0.038	0.003	0.010	0.011	0.019	0.015	0.011	0.013	0.194	0.028	0.202	0.000	0.000

S represents the rhizosphere soil samples from each population sampling plots; R represents the root-endosphere samples from each population sampling plots. Numbers 1–12 represent 12 population sampling plots, which are consistent with the sampling locations listed in **Table 1**. The result of S1 is the mean value of 5 rhizosphere soil samples from sampling site P1, the result of R1 is the mean value of 5 root-endosphere samples from sampling site P1, and the results of the remaining samples are labeled in the same manner.

**Figure 4:**
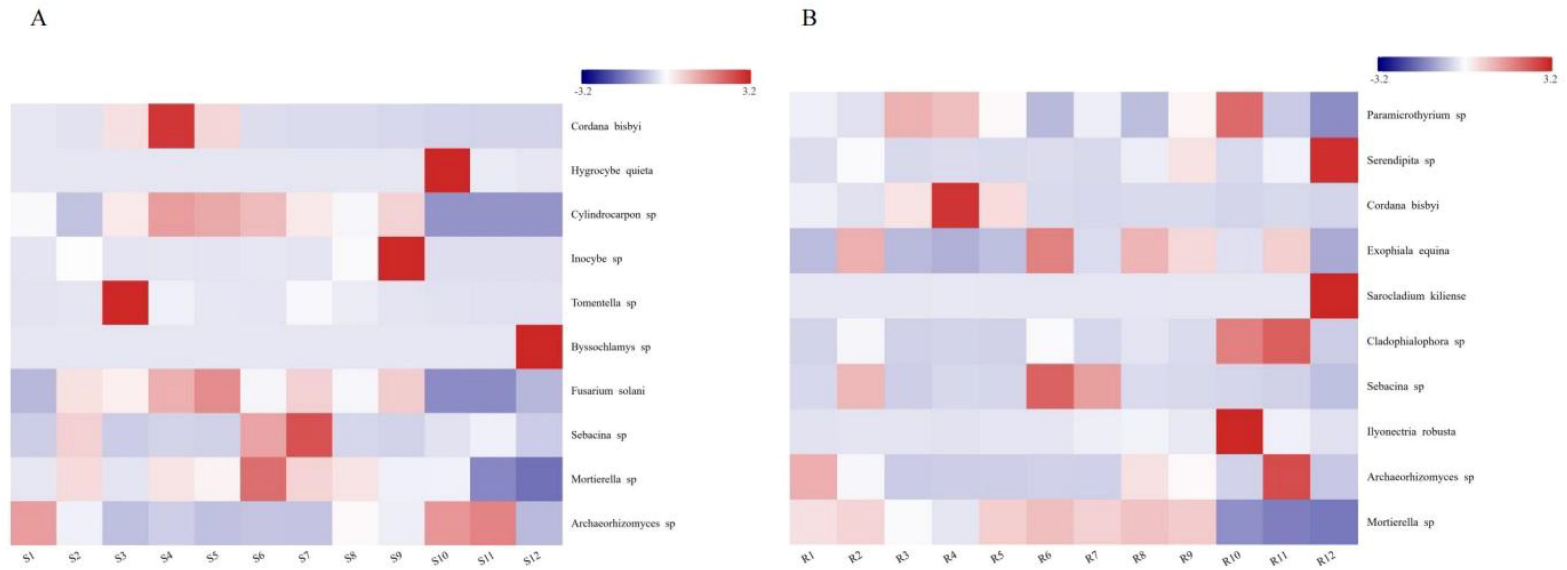
Heatmap of samples from 12 habitats of *C. subtropicum* at species level. **(A)** rhizosphere soil samples from 12 habitats; **(B)** root-endosphere samples of 12 habitats. Based on the ASVs table, the heatmap calculates Z-values based on the relative abundances of the top 10 species at the species taxonomic level using the Bray-Curtis distance algorithm.

Species abundance heatmaps visualize community compositional similarities across samples, where the horizontal axis denotes samples, and the vertical axis represents fungal taxa. The color intensity of each cell corresponds to the Z-score derived from row-wise normalization of relative abundances. Specifically, the Z-score for a given taxon in a sample is calculated as the deviation of the taxon’s relative abundance in that sample from the mean relative abundance of the taxon across all samples, divided by the standard deviation of the taxon’s relative abundance across all samples. A deeper red color indicates a relatively higher abundance of the target taxon in the corresponding sample compared to other samples. Analyses were performed on amplicon sequence variants (ASVs) after excluding unannotated fungal taxa, with only the top 10 most abundant fungal taxa retained at species level (**Figure 4**). The abundance heatmap findings are consistent with the fungal community composition analysis, while providing a more intuitive and detailed visualization of taxon-specific abundance patterns across samples.

### 4.3 Community composition cluster analysis

**Figure 5:**
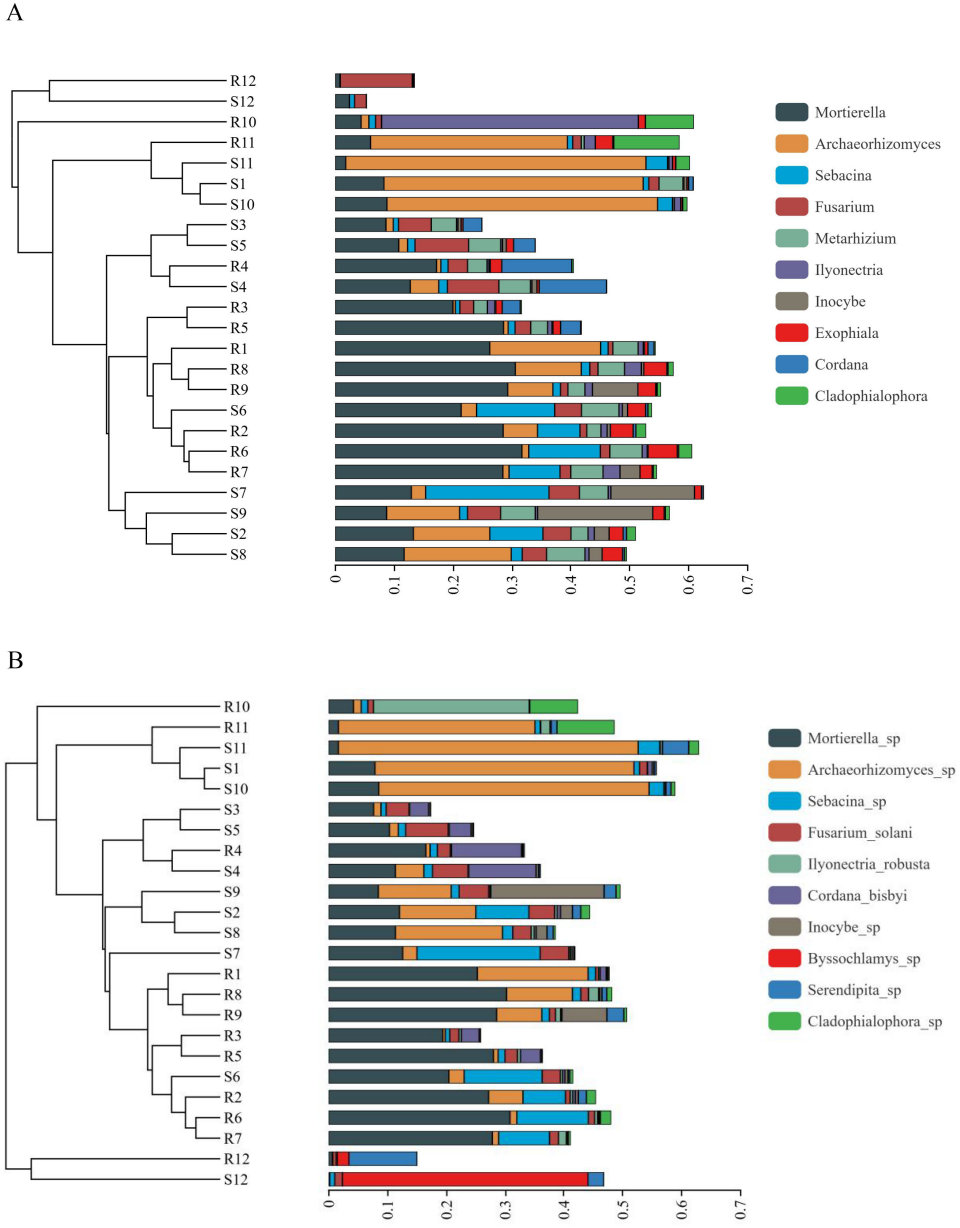
Community composition cluster analysis of root-endosphere and rhizosphere soil samples from 12 habitats of *C. subtropicum*. **(A)** At genus level; **(B)** At species level.

At the genus level, distinct differences in fungal composition were observed between root-endosphere and rhizosphere soil samples of *C. subtropicum*. Notably, the genus *Mortierella* was ubiquitously detected across all samples (both in root-endosphere and rhizosphere soil samples), demonstrating it is widely present in all habitats and may participate in the life activities of *C. subtropicum*, and the dominant fungal species were extremely similar among all samples in the Wenshan area (P1-9). In contrast, *Archaeorhizomyces* exhibited relatively high relative abundances in a subset of both-endosphere and rhizosphere soil samples (S1,S10,S11,R11). At the species level, a subset of the detected fungal species (both in root-endosphere and rhizosphere soil samples) clustered into a single monophyletic clade, reflecting their similarity of the dominant fungal communities composition. However, despite the similar dominant fungi of some taxa, the overall fungal species composition varied markedly across all samples, highlighting strong habitat-specific or sample-type-specific assembly patterns. In terms of abundance, the top fungal taxa with the highest relative abundances were *Mortierella* sp.and *Archaeorhizomyces* sp., which were the core fungal species of *C. subtropicum* in most regions (**Figure 5**).

### 4.4 Alpha diversity analysis

Effective sequences from 120 C*. subtropicum* samples (60 root-endosphere and 60 rhizosphere soil samples) were filtered using Mothur software, and rarefaction curves were plotted based on the Shannon diversity index (**Supplementary Figure 1**). The curves exhibited a distinct plateau phase after the sequencing depth exceeded 24000 reads, indicating that the sequencing data volume was sufficient and the maximum number of amplicon sequence variants (ASVs) had been recovered. The Good’s coverage index reached 1.0 for both root-endosphere and rhizosphere soil samples of *C. subtropicum* (**Supplementary Figure 2**).

**Figure 6:**
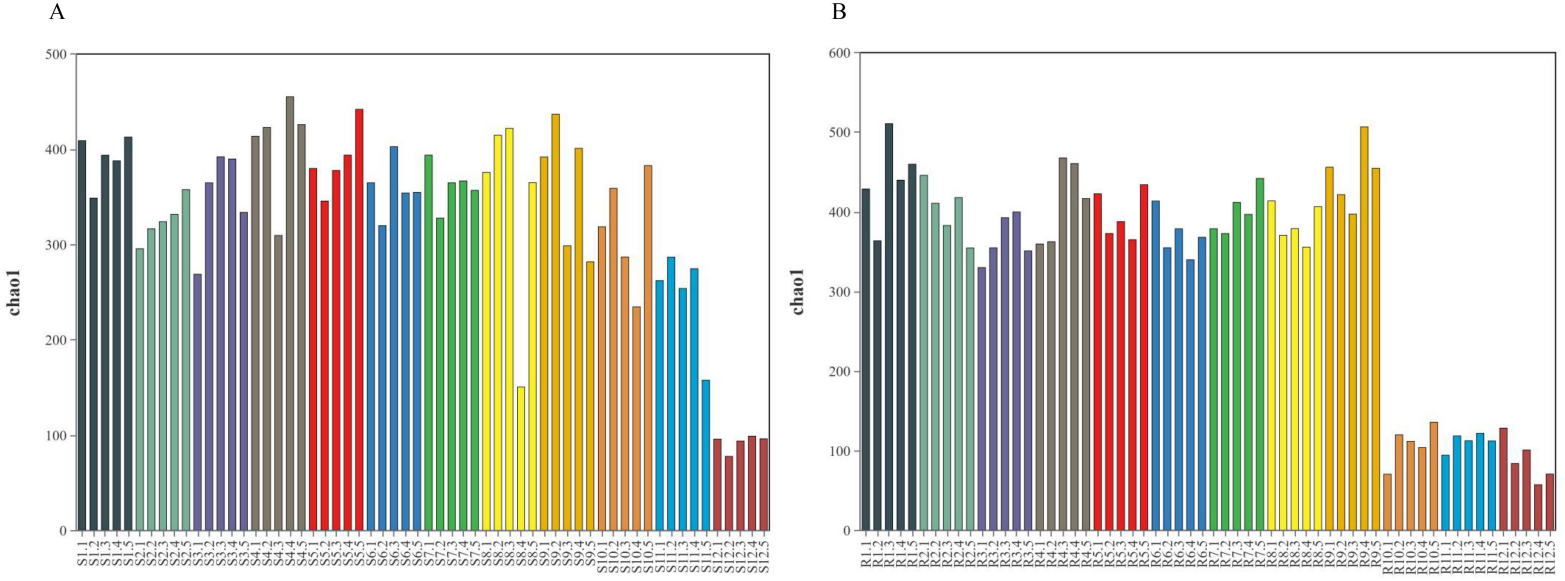
Chao1 index of root-endosphere and rhizosphere soil samples from 12 habitats of *C. subtropicum*. The abscissa is the sample name, and the ordinate is the index average. **(A)** rhizosphere soil samples from 12 habitats; **(B)** root-endosphere samples of 12 habitats.

Among the four main distribution regions, the mean Chao1 index of *C. subtropicum* samples was highest in Wenshan Prefecture (384.32), followed by Baoshan Prefecture (212.88), Nujiang Prefecture (179.96), and Kunming (90.87). The comparison results of Chao1 diversity index can be intuitively observed from **Figure 6**. The mean Shannon-E index values for the root-associated samples of four regions were 4.27 (Wenshan), 3.17 (Baoshan), 3.26 (Nujiang), and 2.70 (Kunming), while the mean Simpson index values were 0.059 (Wenshan), 0.15 (Baoshan), 0.11 (Nujiang), and 0.17 (Kunming), respectively (**Supplementary Table 4**). These results collectively indicated that root-associated samples of Wenshan Prefecture harbored the highest fungal communities diversity (characterized by the richest species pool and most uniform abundance distribution), whereas root-associated samples of Kunming exhibited the lowest diversity. For *C. subtropicum* populations with distinct life histories, the mean Chao1 index was 387.75 for root-associated samples of wild populations in Malipo County and 381.58 for introduced populations in the same region, showing negligible differences between the two groups. This similarity was attributed to the fact that introduced *C. subtropicum* (planted in 2009, 2018, and 2022) were grown in unmanaged wild habitats of Malipo County, and had acclimated to the local environment after long-term adaptation. In contrast, root-associated samples of seed-propagated cultivated seedlings in Kunming, whose seeds were derived from artificial pollination of the wild populations of *C. subtropicum* in Malipo County, had relatively low mean Chao1 index (90.87). The mean Shannon-E indices values for root-associated samples of wild, introduced, and cultivated populations were 4.25, 4.28, and 2.70, while the mean Simpson index were 0.065, 0.054, and 0.17, respectively (**Supplementary Table 5**). The results showed that for the root-associated samples of wild populations, whether native populations or introduced populations, the diversity of their root-associated fungal communities is higher than that of cultivated populations (Kunming). The boxplot of intergroup difference analysis can intuitively reflect the median, dispersion degree, maximum value, minimum value, and outliers of species diversity within each sample group. The results of intergroup difference analysis of diversity indices of root-endosphere and rhizosphere soil samples in 12 habitats are presented in **Supplementary Figure 3**. Kruskal-Wallis rank sum tests revealed significant intergroup differences in all three diversity indices: Chao1 (P = 8.6e^-09^), Shannon-E (P = 1.636e^-06^) and Simpson (P = 0.00025).

### 4.5 Beta-diversity analysis

**Figure 7:**
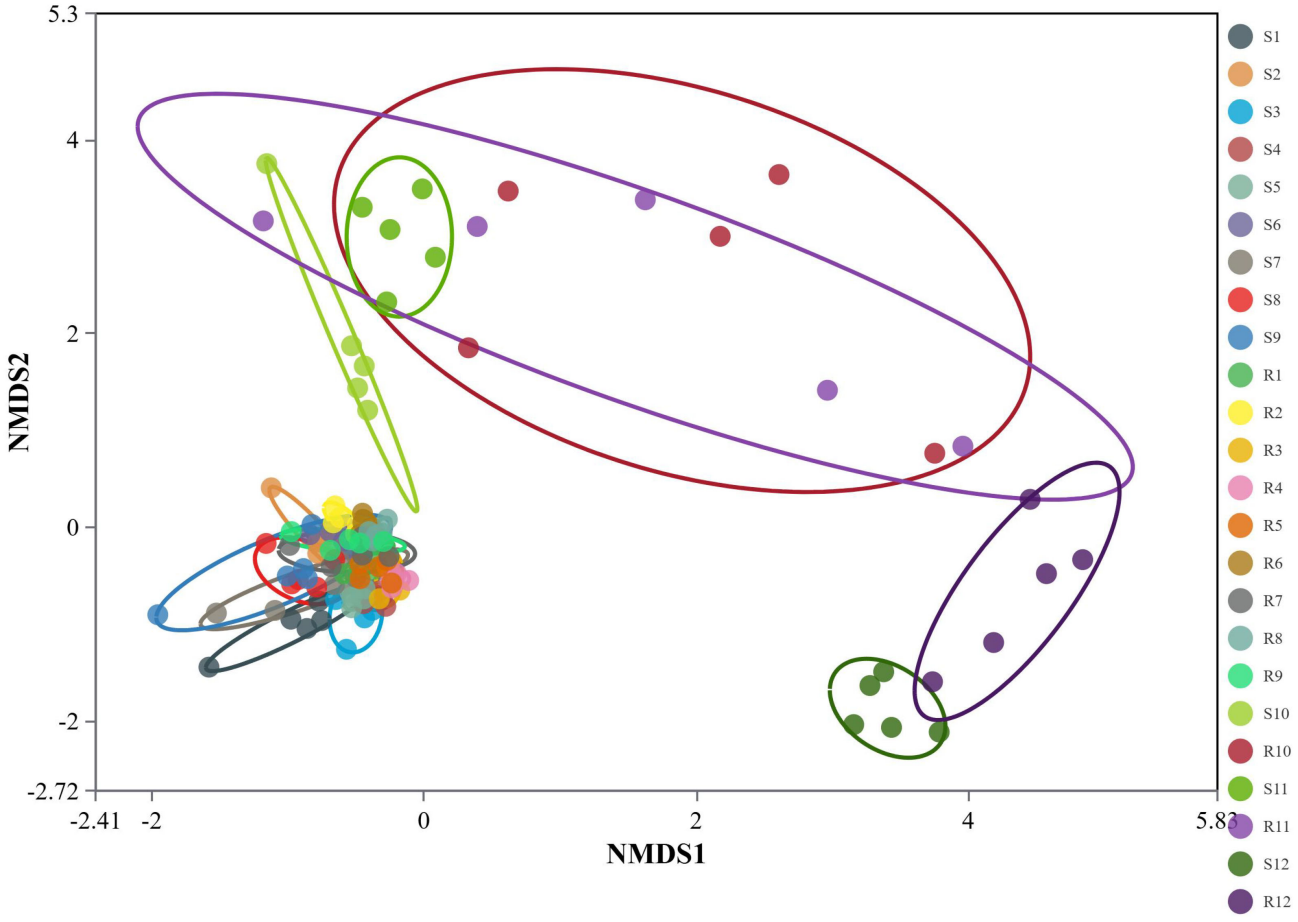
NMDS analysis of root-endosphere and rhizosphere soil samples from 12 habitats of *C. subtropicum*. Points of different colors or shapes represent samples; the horizontal and vertical axes indicate values based on quantitative distance matrices. The closer the spatial distance between samples, the more similar their species composition and community structure. The stress value was 0.14 and the results were calculated using the Bray-Curtis distance algorithm.

NMDS particularly suitable for analyzing the fungal community data of *C. subtropicum*, which encompasses multiple populations, diverse habitat types, and a broad range of fungal taxa. NMDS ordination results revealed distinct spatial clustering patterns of *C. subtropicum* samples (root-endosphere and rhizosphere soil) based on their geographic origin. Specifically, sample points corresponding to both root-endosphere and rhizosphere soil samples from Wenshan Prefecture (P1-P9) exhibited pronounced aggregation, whereas samples from Longling County (Baoshan Prefecture, P10), Gongshan County (Nujiang Prefecture, P11), and Kunming (P12) were relatively dispersed in the ordination space, and the stress value was 0.14 (**Figure 7**). The composition and structure of the fungal community in the root-associated samples of *C. subtropicum* in the Wenshan area are more similar. The results of the significance analysis of inter-group community structure differences are presented in **Supplementary Table 6**, including PERMANOVA R² and P value.

### 4.6 Co-occurrence network analysis

**Figure 8:**
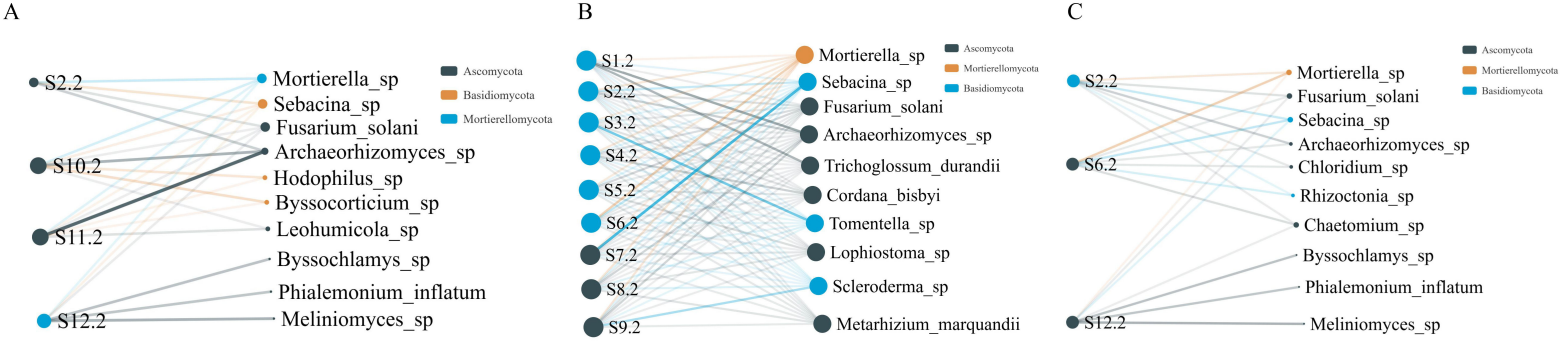
Co-occurrence network diagram. Co-occurrence relationship of species in different samples is visually displayed. Nodes in the network represent sample nodes or species nodes, and the connection between sample nodes and species nodes represents the sample containing the species. **(A)** rhizosphere soil samples of *C. subtropicum* in 4 major geographical regions: Wenshan, Baoshan, Nujiang, and Kunming; **(B)** different habitats in Wenshan different areas; **(C)** rhizosphere soil samples of *C. subtropicum* in 3 life history types (wild, introduced, and seed-propagated cultivated).

To elucidate the co-occurrence patterns of fungi across varying geographic regions and population types of *C. subtropicum*, co-occurrence network analysis was exclusively performed on rhizosphere soil samples of this orchid species. At the species taxonomic level, the analysis was restricted to the top 10 fungal taxa with the highest richness, as derived from the amplicon sequence variant (ASV) abundance table, to construct the co-occurrence network. The higher the relative abundance of species in the samples, the thicker and more obvious the connecting lines between them. A species-level collinearity network analysis diagram was constructed from the rhizosphere soil samples of *C. subtropicum* in four major geographical regions: Wenshan, Baoshan, Nujiang, and Kunming. The results showed that *Mortierella* sp., *Fusarium solani*, and *Sebacina* sp. were distributed in all four regions. *Phialemonium inflatum*, *Byssochlamys* sp., and *Meliniomyces* sp. were only distributed in the environmental samples of Kunming. In the Wenshan area, where the diversity of the root-associated fungal community of *C. subtropicum* is the highest, the 10 fungal species with the highest abundance are distributed in both the environmental samples of wild populations and introduced populations. The difference is that the abundance of fungi contained in each sample varies. For *C. subtropicum* populations with distinct life history types (wild, introduced, and seed-propagated cultivated), *Mortierella* sp., *Fusarium solani*, *Chaetomium* sp. and *Sebacina* sp. were identified as common taxa in rhizosphere soil samples of 3 life history types and *Phialemonium inflatum*, *Byssochlamys* sp., and *Meliniomyces* sp. were only distributed in cultivated rhizosphere soil samples (**Figure 8**). The overlap of these taxa across population types suggests their adaptability to both natural and managed growth conditions, and their potential importance for the survival and establishment of *C. subtropicum* across different propagation modes.

## 5 Discussion

Root-associated fungal communities, including those in the root-endosphere and rhizosphere soil, play a crucial role in orchid growth, nutrient uptake, stress tolerance, and habitat acclimation, and are involved in key life cycle processes (Waterman et al., 2011; Rasmussen et al., 2015; Lin et al., 2020; Tedersoo et al., 2020; Zhao et al., 2021; Gao et al., 2022; Liang et al., 2022). Root-endosphere fungi include fungi on the root surface and within root tissues. Some fungi, such as mycorrhizal fungi, from root-associated fungi of Orchidaceae enhance nutrient uptake, regulate plant hormones, and mitigate environmental stress, promoting seedling establishment and population persistence (Liu et al., 2010; Gao et al., 2020; Zhang et al., 2020; Li et al., 2021a; Zettler and Dvorak, 2021; Chen et al., 2022; Li et al., 2022; Liao et al., 2025). Clarifying their diversity, structure, and growth-promoting effects is essential for optimizing *ex situ* conservation and artificial propagation of this flagship endangered orchid, a core strategy for alleviating extinction risks of endangered orchids (Salazar-Cerezo et al., 2018; Dearnaley et al., 2012; Zhou et al., 2023; Abeli et al., 2020; Ren et al., 2021; Kaushik et al., 2021; Li et al., 2024). A growing body of evidence indicates that orchid mycorrhizal fungi (OMF) are widely distributed in both orchid mycorrhizae and surrounding soils, with most root-associated OMF also present in soil environments (Bonnardeaux et al., 2007; Lin et al., 2020; Jacquemyn et al., 2021; Wang et al., 2021). The research on diversity and composition of root-associated fungal communities is critical for the restoration of endangered orchid populations and the implementation of artificially assisted colonization programs (Cribb et al., 2003; Favre-Godal et al., 2020; Bell et al., 2020; Liu et al., 2020; Li et al., 2021b; Han et al., 2021).

*Cypripedium* (lady’s slipper orchids) is an ecologically and evolutionarily important lineage within Orchidaceae, and *C. subtropicum* stands out as a flagship species of this genus due to its narrow distribution and high conservation value. To address the knowledge gap for *C. subtropicum*, the present study collected root-endosphere and rhizosphere soil samples from different regions, habitats, and population types (wild, introduced, cultivated) in Yunnan Province, and employed Illumina Novaseq high-throughput sequencing to detect and characterize the diversity and structure of root-associated fungal communities of *C. subtropicum*. Significant regional differences in fungal diversity were observed, consistent with the results: Wenshan Prefecture had the highest fungal richness (34,564 ASVs, 87.76% of total), followed by Baoshan, Nujiang, and Kunming. This pattern aligns with prior findings that habitat stability and heterogeneity shape fungal community diversity (Li et al., 2021a; Ren et al., 2021), as Wenshan’s stable subtropical habitat supports diverse fungal assemblages that likely benefit *C. subtropicum* by enhancing nutrient cycling and microhabitat adaptability. In contrast, Kunming’s cultivated populations had the lowest diversity (average 90 ASVs per sample), likely due to the homogenized artificial environment, which may limit plant growth potential.

*C. subtropicum* has a narrow distribution range, occurring in Yunnan, Guangxi, and Tibet (China) as well as Vietnam. Wild populations in Yunnan are scattered at elevations of 1354–1506 m, with most populations having small population sizes. This unique niche makes it an irreplaceable model for investigating the conservation biology of subtropical endangered orchids. Introduced plants can survive in wild habitats at 1157–1200 m, and seed-propagated individuals thrive in greenhouses at 1935 m, demonstrating moderate elevation adaptability. The similarities and differences of fungi communities in root-endosphere and rhizosphere soil samples of *C. subtropicum* in different habitats were analyzed in order to find out possible important fungi species. Ascomycota, Basidiomycota, and Mortierellomycota were dominant phyla, with *Mortierella*, *Archaeorhizomyces*, *Sebacina*, and *Fusarium* as core genera, showing distinct regional specificity: *Mortierella* dominated Wenshan, *Archaeorhizomyces* Nujiang, *Ilyonectria* Baoshan, and *Fusarium* Kunming’s cultivated samples. *Mortierella* (42.5%-68.3% in Wenshan root-endosphere) likely promotes root development and nutrient availability, consistent with its known ecological roles in orchid root-associated fungal interactions (Gang et al., 2017; Fan et al., 2024), while Fusarium’s high abundance in Kunming warrants further investigation into its growth impacts.

NMDS and cluster analysis showed Wenshan samples aggregated closely (indicating conserved fungal communities), while other regions’ samples were dispersed—reflecting a stable co-occurrence in Wenshan’s core habitat. Co-occurrence network analysis identified three pan-regional core species (*Mortierella* sp., *Fusarium solani*, *Sebacina* sp.) critical for supporting *C. subtropicum* across habitats, while *Phialemonium inflatum* and *Byssochlamys* sp. were exclusive to Kunming’s cultivated samples, reflecting artificial environment This aligns with studies showing that environmental filtering drives fungal community differentiation across habitats (Li et al., 2021b; Lin et al., 2020). Wild and introduced populations in Malipo (Wenshan) had similar fungal diversity, due to introduced populations’ acclimation to local wild habitats-key for *ex situ* conservation success, as native fungal communities enhance orchid adaptability (Favre-Godal et al., 2020; Bell et al., 2020). Kunming’s cultivated populations had significantly lower diversity, suggesting artificial practices disconnecting plants from native microbes compromise symbiosis and growth performance, consistent with prior findings on cultivated orchid populations (Swarts and Dixon, 2009; Xing et al., 2020). These findings inform *C. subtropicum* conservation: Wenshan is the most suitable habitat, and protecting it is critical for maintaining fungal symbionts. *Ex situ* conservation should prioritize introducing plants into local wild habitats (e.g., Malipo), while artificial propagation could benefit from inoculating seedlings with core fungal species (*Mortierella* sp., *Sebacina* sp.) to improve survival (Moreno-Camarena and Ortega-Larrocea, 2022; Tian et al., 2018).

A key distinction from prior studies on *Cypripedium* species is the absence of *Tulasnella*-a typical dominant mycorrhizal genus for most *Cypripedium* species-in *C. subtropicum*, particularly that it is not among the top 10 most abundant fungal genera in the species’ root-endosphere and rhizosphere soil samples (Shefferson et al., 2005; Miao et al., 2015; Xu et al., 2019). This discrepancy can be attributed to *C. subtropicum*’s unique habitat niche: as the only *Cypripedium* species endemic to Asian subtropical forests, its wild populations in Yunnan Province are restricted to a narrow elevation range of 1354–1506 m, which differs drastically from the cool-temperate or alpine environments occupied by most other *Cypripedium* species, as previous investigations into *Cypripedium* mycorrhizal symbiosis have predominantly focused on species inhabiting such temperate, alpine, or boreal ecosystems (Miao et al., 2015; Xu et al., 2019; Weerasuriya et al., 2022), with rare systematic studies on taxa adapted to subtropical evergreen broad-leaved forests-the only natural habitat of *C. subtropicum*. As the first comprehensive characterization of fungal diversity and community structure across multiple biological compartments (root-endosphere and rhizosphere soil) of *C. subtropicum*, this study bridges the taxonomic and habitat-specific gap in *Cypripedium* fungal diversity and community structure research, identifying *Mortierella*, *Archaeorhizomyces*, and *Sebacina* as the dominant fungi instead of the typically core genus *Tulasnella* associated with temperate *Cypripedium* species (Miao et al., 2015; Xu et al., 2019). This challenges the long-standing paradigm of T*ulasnella* predominance in *Cypripedium* fungi, suggesting that *Cypripedium* fungi has undergone adaptive radiation in response to subtropical environmental conditions (e.g., higher soil humidity, year-round moderate temperatures, and distinct soil microbial pools), which has driven the selection of a distinct suite of fungal communities, displacing *Tulasnella* from its typical dominant role, enriching both the evolutionary framework of orchid-fungal mutualism across global biomes and our knowledge of subtropical orchid fungal assembly (Li et al., 2024; Lin et al., 2020).

## 6 Conclusion

This study investigated the diversity and composition of root-associated fungal communities (root-endosphere and rhizosphere soil) of *C. subtropicum* across four geographical regions (Wenshan, Baoshan, Nujiang, Kunming) in Yunnan Province, covering different population types. High-throughput sequencing and diversity analyses confirmed that regional differences in fungal diversity were significant. Wenshan Prefecture had the highest ASV richness (34,564 ASVs, 87.76% of the total) and alpha diversity (mean Chao1: 384.32, Shannon-E: 4.27), while Kunming had the lowest (mean Chao1: 90.87, Shannon-E: 2.70). Baoshan and Nujiang showed intermediate diversity. Taxonomically, Ascomycota, Basidiomycota, and Mortierellomycota were dominant phyla, with *Mortierella*, *Archaeorhizomyces*, *Sebacina*, and *Fusarium* as core genera. Dominant taxa varied by region. *Mortierella* dominated in Wenshan, *Archaeorhizomyces* in Nujiang, *Ilyonectria* in Baoshan, and *Fusarium* in Kunming’s cultivated samples. NMDS and cluster analysis showed Wenshan samples aggregated closely, indicating conserved fungal communities, while other regions’ samples were dispersed. Co-occurrence network analysis identified *Mortierella* sp., *Fusarium solani*, and *Sebacina* sp. as pan-regional core species, while *Phialemonium inflatum* and *Byssochlamys* sp. were exclusive to Kunming’s cultivated samples. Wild and introduced populations in Wenshan had similar fungal diversity, while Kunming’s cultivated populations had lower diversity. Geographic region, habitat, and population type are key factors shaping fungal communities. Wenshan provides optimal habitat for *C. subtropicum* and its fungi, while artificial cultivation in Kunming reduces fungal diversity. Core fungal species could aid *C. subtropicum* conservation and artificial propagation.

## Data Availability

The datasets presented in this study can be found in online repositories. The names of the repository/repositories and accession number(s) can be found in the article/**Supplementary Material**.
