# Supplementary figures and images for: Diversity and composition of root-associated fungal communities in critically small population of Cypripedium subtropicum
Source: Front Plant Sci. 2026 Jun 3;17:1783975. doi: 10.3389/fpls.2026.1783975 (PMC13272303; doi:10.3389/fpls.2026.1783975)

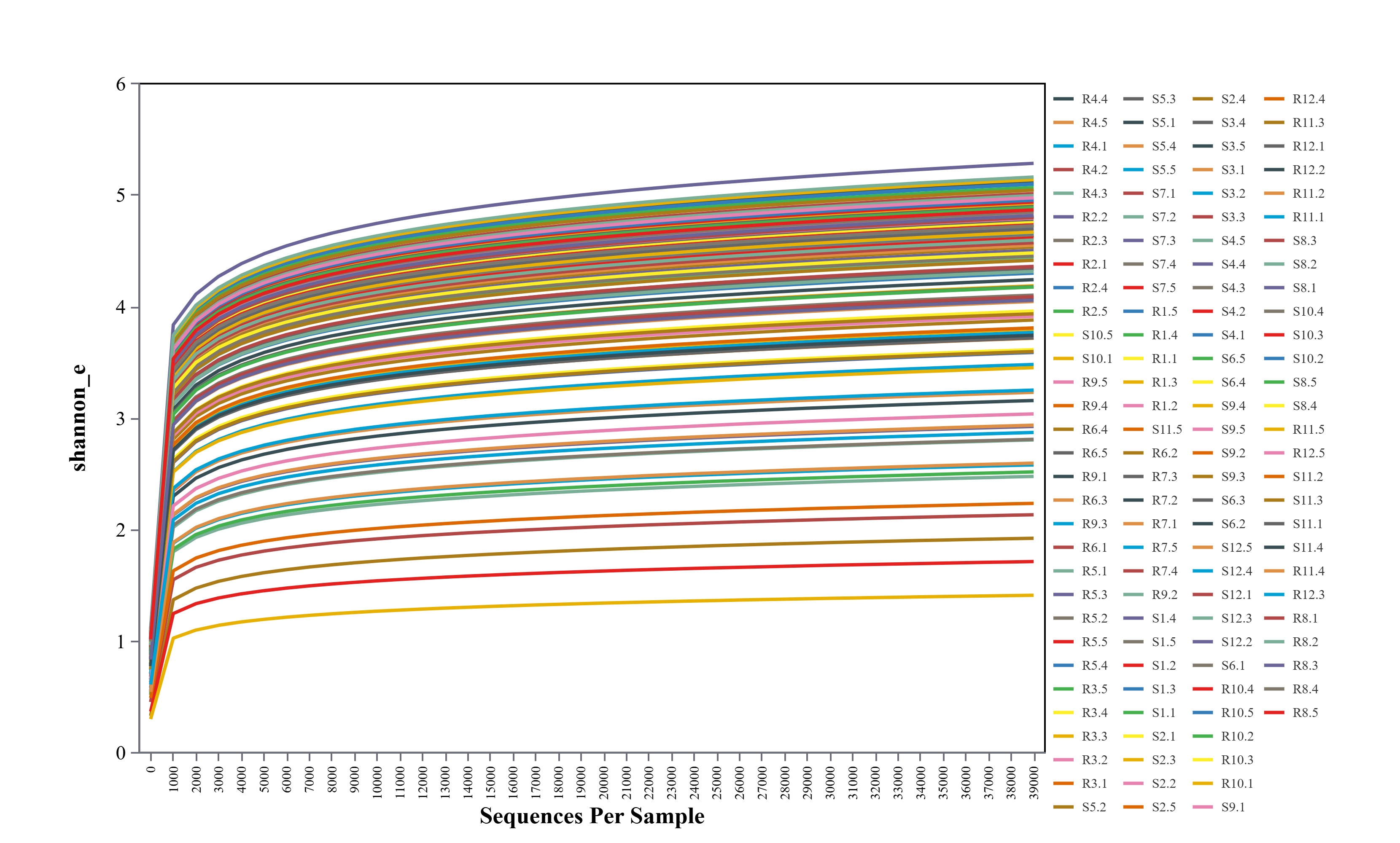

Supplement: Supplementary file 1 [file Image1.jpeg]

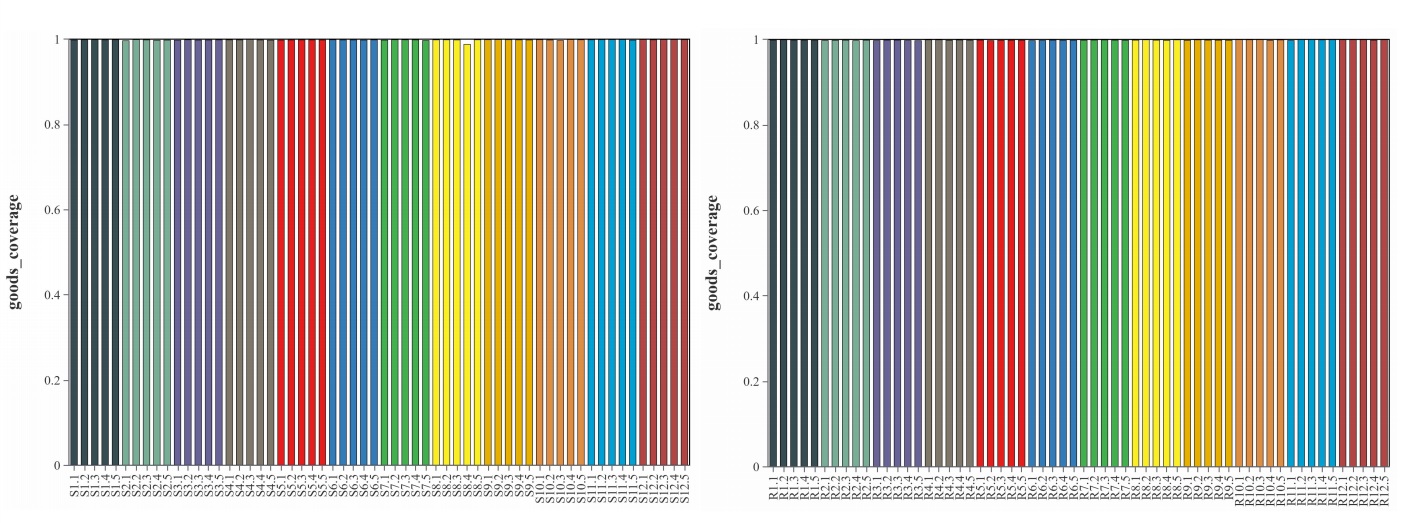

Supplement: Supplementary file 2 [file Image2.jpeg]

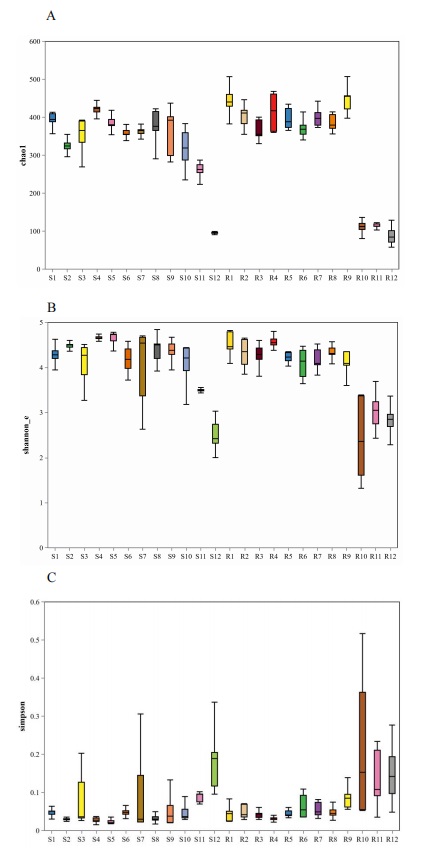

Supplement: Supplementary file 3 [file Image3.jpeg]
